# Final destination: Senescence—NtNAC56 and jasmonic acid in the regulation of leaf senescence in tobacco

**DOI:** 10.1093/plphys/kiae179

**Published:** 2024-03-21

**Authors:** Pablo Ignacio Calzadilla

**Affiliations:** Assistant Features Editor, Plant Physiology, American Society of Plant Biologists; Instituto de Fisiología Vegetal (INFIVE), Universidad Nacional de La Plata—CONICET, cc 327, 1900 La Plata, Buenos Aires, Argentina; Department of Earth and Environmental Sciences, Faculty of Science and Engineering, University of Manchester, Manchester M13 9PT, UK

Leaf senescence is the timely coordinated program through which leaf tissue dies. This program is the last stage of leaf development, when nutrients are recycled and mobilized to sink tissues. Senescence triggers leaf chlorosis (“yellowing”), the accumulation of reactive oxygen species (ROS), and the disassembly of the photosynthetic apparatus (Krieger-Liszkay et al. 2019). The latter involves chloroplast dismantling, leading to chlorophyll loss and thylakoid membrane reorganization (Domínguez and Cejudo 2021). Thus, senescence reduces the overall photosynthetic capacity of the leaf. Due to its impact on leaf physiology and the productivity of the whole plant, efforts have been made to understand the molecular signals regulating leaf senescence (Zhou and Yang 2023). Delaying senescence, particularly under stress conditions, can increase plant yield and resilience, relevant traits for crop improvement in the current climate change scenario.

Senescence can be induced by developmental and environmental cues and is regulated by different endogenous signals. In particular, NAC transcription factors (TFs) regulate leaf senescence, inducing the expression of SENESCENCE-ASSOCIATED GENES (*SAG*) (Kim et al. 2016) and phytohormones responses (Zhou et al. 2013; Sakuraba et al. 2020). Within the phytohormone-related responses, jasmonic acid (JA) is a known senescence mediator (Ullah et al. 2019), being the expression of its biosynthetic genes upregulated during this developmental program (He et al. 2002). Although myriad endogenous factors control leaf senescence (Zhou and Yang 2023), the integration and coordination of these signals remain unclear.

In this Issue of *Plant Physiology*, Chang et al. (2024) studied the connection between NAC TFs and JA in regulating leaf senescence in tobacco plants. Exploring previous transcriptome data, the authors identified NtNAC56 as a candidate regulatory TF for leaf senescence. To study NtNAC56's role, transgenic lines overexpressing *NtNAC56* (*NtNAC56-*OE) were generated and characterized. The *NtNAC56*-OE lines senesced earlier than the wild-type (WT) plants. *NtNAC56*-OE plants had significantly lower chlorophyll content and photosynthetic capacity and higher expression of SAGs compared to WT.

Since senescence triggers chloroplast dismantling, the authors analyzed the effect of *NtNAC56* overexpression on chloroplast ultrastructure using transmission electron microscopy. *NtNAC56*-OE lines showed smaller chloroplasts, with fewer grana stacks and starch granules. This phenotype was also associated with cellular redox imbalance, evidenced by altered expression of antioxidant enzyme genes and an increased malondialdehyde content. Thus, *NtNAC56* overexpression triggers chloroplast degradation and ROS accumulation in tobacco leaves, supporting its positive regulatory role in leaf senescence.

Although the role of NtNAC56 as a senescence-inducing factor was confirmed, its molecular signaling pathway remained unknown. To address this issue, Chang et al. (2024) performed an RNA-seq analysis comparing 8-week-old leaves of NtNAC56-OE and WT tobacco plants. A total of 7,917 differentially expressed genes were identified: 4,231 and 3,686 upregulated and downregulated, respectively. A gene ontology enrichment analysis indicated “response to JA stimulus” as one of the most highly enriched in the upregulated group. JA has previously been reported to promote leaf senescence and increase the expression of *NAC TFs* genes (Zhu et al. 2015). Therefore, the authors studied the effect of JA on NtNAC56 expression using methyl jasmonate (MeJA). The addition of MeJA was evaluated on transgenic tobacco plants expressing the β-GLUCURONIDASE (GUS) reporter gene downstream of the *NtNAC56* promoter (*NtNAC56pro::GUS*). MeJA increased GUS staining of the *NtNAC56pro::GUS* leaves, confirming JA activation of *NtNAC56* expression.

Once induced, NtNAC56 will activate a downstream gene regulatory network, triggering leaf senescence. To explore the NtNAC56-induced signaling cascade, the authors searched for its target genes. A DNA affinity purification sequencing was performed, identifying 1,300 enriched NtNAC56 genome binding sites. Among them, 6 DNA motifs shared a specific core binding motif: TTTCTT. Interestingly, the promoter of *NtLOX5*, a gene involved in JA biosynthesis, contained this TTTCTT-binding motif. The interaction between NtNAC56 and the *NtLOX5* promoter was studied by a dual-luciferase reporter assay, cloning the *NtLOX5* promoter upstream of the luciferase (*LUC*) reporter gene (*NtLOX5pro::LUC*). The presence of NtNAC56 increased LUC expression, confirming NtNAC56 activation of the *Nt*LOX5 promoter likely via the TTTCTT-binding motif. Regulation of NtLOX5 supports the higher JA content observed in the *NtNAC56* overexpressing lines.

In summary, Chang et al. (2024) used a broad variety of techniques to explore the regulatory mechanisms inducing leaf senescence in tobacco. They identified NtNAC56 as a novel TF contributing to JA-induced leaf senescence. Data showed a positive activation loop between NtNAC56 and JA and that NtNAC56 directly regulates JA biosynthesis through its binding to the specific TTTCTT *cis*-regulatory motif (Fig. 1). Overall, this study contributes to our knowledge of the complex signaling network regulating leaf senescence. Understanding these regulatory pathways will pave the way for manipulating senescence in plants, an important trait toward crop improvement in the era of climate change.

**Figure 1.**
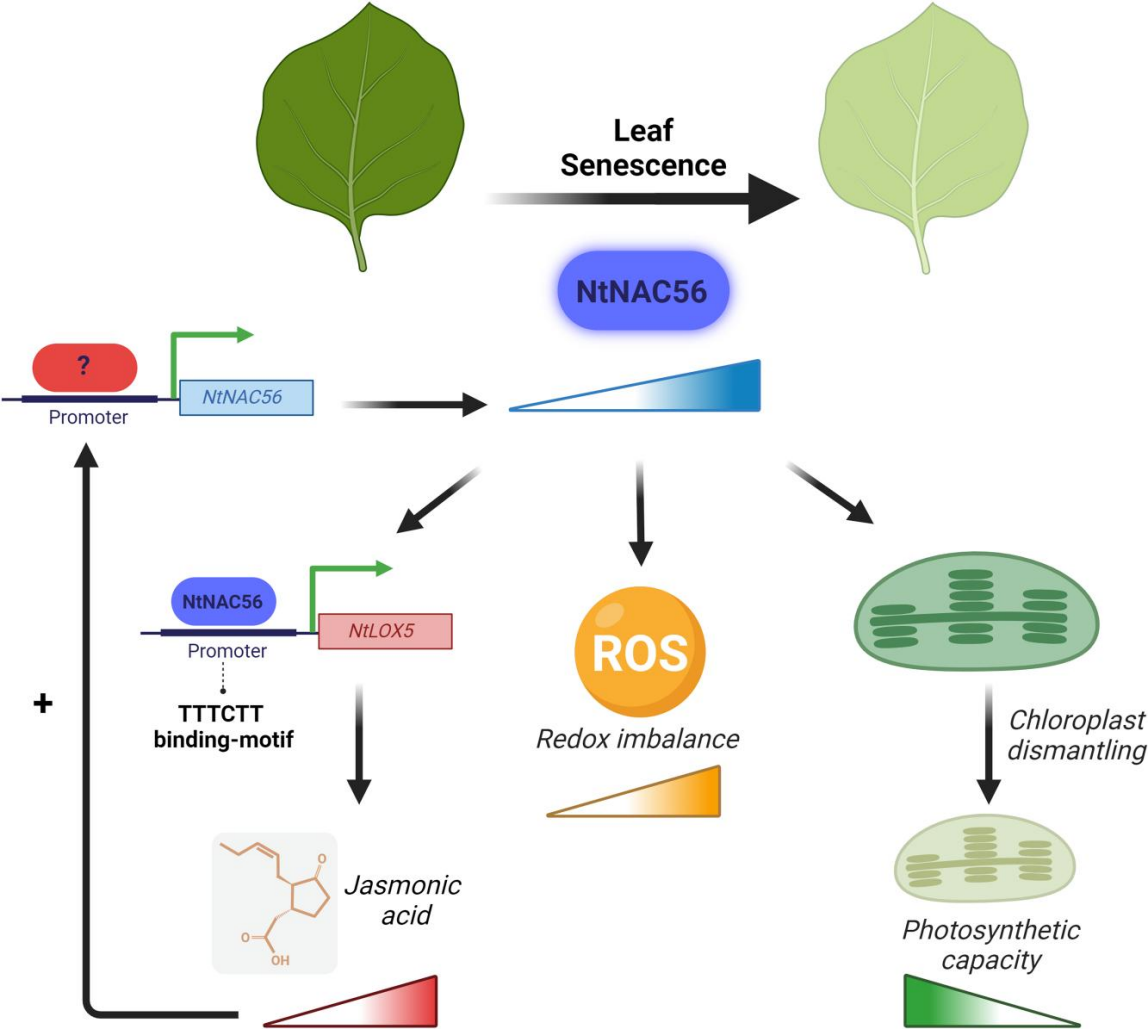
The transcription factor NtNAC56 induces leaf senescence in tobacco. The accumulation of NtNAC56 triggers redox homeostasis imbalance, a decrease in the photosynthetic capacity of the leaf and increased JA content. NtNAC56 regulates *NtLOX5* expression, a gene participating in JA biosynthesis, likely through binding to a TTTCTT motif in the *NtLOX5* promoter region. JA accumulation also induces *NtNAC56* expression, generating a positive regulatory loop for leaf senescence. The molecular pathway through which JA activates *NtNAC56* expression remains unknown. Created with BioRender.com.

## Dive Curated Terms

The following phenotypic, genotypic, and functional terms are of significance to the work described in this paper:

NAC CHEBI: CHEBI:7421

leaf AmiGo: PO:0025034

leaf senescence Planteome: TO:0000249
